# Supplementary material for: Metabolome and Transcriptome Reveal the Regulatory Mechanism of Anthocyanin Synthesis in Tuber Skin and Flesh of Dioscorea alata L
Source: Plants (Basel). 2025 Nov 12;14(22):3454. doi: 10.3390/plants14223454 (PMC12655603; doi:10.3390/plants14223454)
Supplement: Supplementary file 1 [file plants-14-03454-s001.zip › Supplementary tables and figures.pdf]

Table S1 – List of primers used for qRT-PCR assays.

Gene ID	Primer ID	Sequences (5' to 3')
Dioal.14G003800.1	*DaPAL*-F	GGATGAGCTAAAGGCCGTGT
	DaPAL-R	GCTCTTCCCTCACAAACCGA
Dioal.07G099300.1	*DaC4H*-F	GGGCATTGCTGAGCTAGTGA
	DaC4H-R	CAGCTTGAAGGTAGGGGAGC
Dioal.16G050500.1	*Da4CL-F*	TTGCATCCCATTCTCCACCC
	Da4CL-R	TGTTGTGCGACGCTAGTGAT
Dioal.10G004500.1	*DaCHS*-F	GAAGGATGTGCCAGGGTTGA
	DaCHS-R	TAGCAGGTCCACCAGGATGA
Dioal.15G024900.1	*DaF3H*-F	GGATCTTGTGCTCGGAGTCG
	DaF3H-R	AGGAGTTGGGAGTTGGCTTG
Dioal.18G078500.1	*DaF3'H*-F	CTGTTGACATGGCCAAAGCC
	DaF3'H-R	GACCGTAAGGGGACCAAGTG
Dioal.09G028200.1	*DaF3'5'H*-F	GGGACAACTCTGTCGCTGAA
	DaF3'5'H-R	TGCCCACTCTATGGTGCTTG
Dioal.04G138500.1	*DaANS*-F	GGCAACCAAGGTGTTCGTTC
	DaANS-R	GATGGTCTCGCTCTGACTCG
Dioal.18G064100.1	*DaEF-1a*-F	TCAGGCTGACTGTGCTGTCCT
	DaEF-1a-R	GTGGTGGCGTCCATCTTGTT

Table S2. RNA-seq data and quality control information.

Sample	Raw reads	Raw bases	Clean reads	Clean bases	Q20_rate	Q30_rate	GC_content
ppf1	43,390,278	6,508,541,700	43,204,712	6,203,498,719	99%	95%	45%
ppf2	43,380,706	6,507,105,900	43,220,842	6,189,540,197	99%	96%	45%
ppf3	43,384,336	6,507,650,400	43,216,078	6,173,008,099	99%	95%	45%
pps1	43,392,904	6,508,935,600	43,255,612	6,217,158,420	99%	96%	45%
pps2	43,357,448	6,503,617,200	43,223,144	6,237,808,780	99%	96%	45%
pps3	43,376,656	6,506,498,400	43,237,828	6,251,430,830	99%	96%	45%
pwf1	43,380,506	6,507,075,900	43,201,594	6,194,999,415	99%	95%	45%
pwf2	43,398,436	6,509,765,400	43,229,886	6,187,769,366	99%	95%	45%
pwf3	43,348,604	6,502,290,600	43,188,774	6,208,389,975	98%	95%	45%
pws1	43,345,418	6,501,812,700	43,220,008	6,140,922,399	99%	96%	45%
pws2	43,364,016	6,504,602,400	43,248,080	5,992,864,449	99%	96%	46%
pws3	43,363,880	6,504,582,000	43,238,274	6,120,972,996	99%	96%	45%
wwf1	43,353,220	6,502,983,000	43,189,970	6,164,913,940	99%	96%	45%
wwf2	43,368,098	6,505,214,700	43,201,708	6,186,247,137	99%	95%	45%
wwf3	43,342,558	6,501,383,700	43,184,984	6,210,635,618	99%	96%	45%
wws1	43,371,600	6,505,740,000	43,248,094	6,088,574,338	99%	96%	46%
wws2	43,352,148	6,502,822,200	43,215,912	6,182,047,956	99%	96%	46%
wws3	43,380,932	6,507,139,800	43,233,908	6,230,289,623	99%	95%	46%

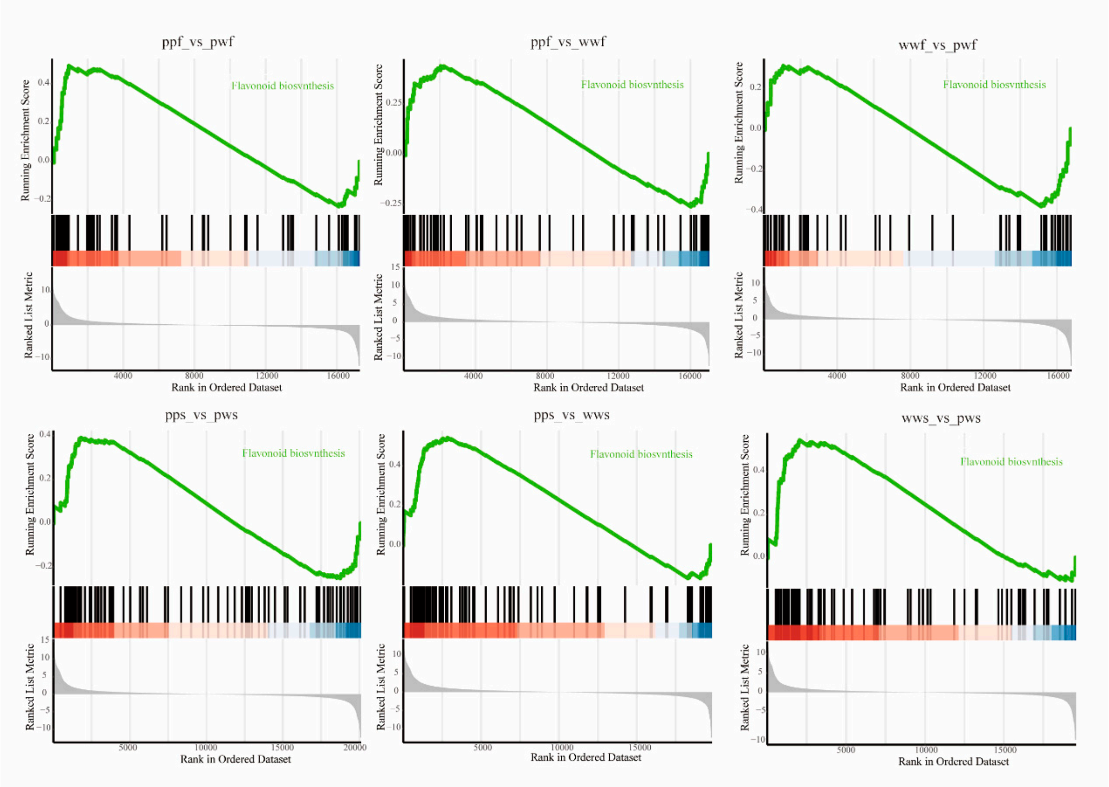

Figure S1. GSEA (gene set enrichment analysis) of flavonoid biosynthetic process genes. GSEA rank was calculated by ppf _vs_pwf, ppf_vs_wwf, wwf_vs_pwf, pps_vs_pws, pps_vs_wws, wws_vs_pws.

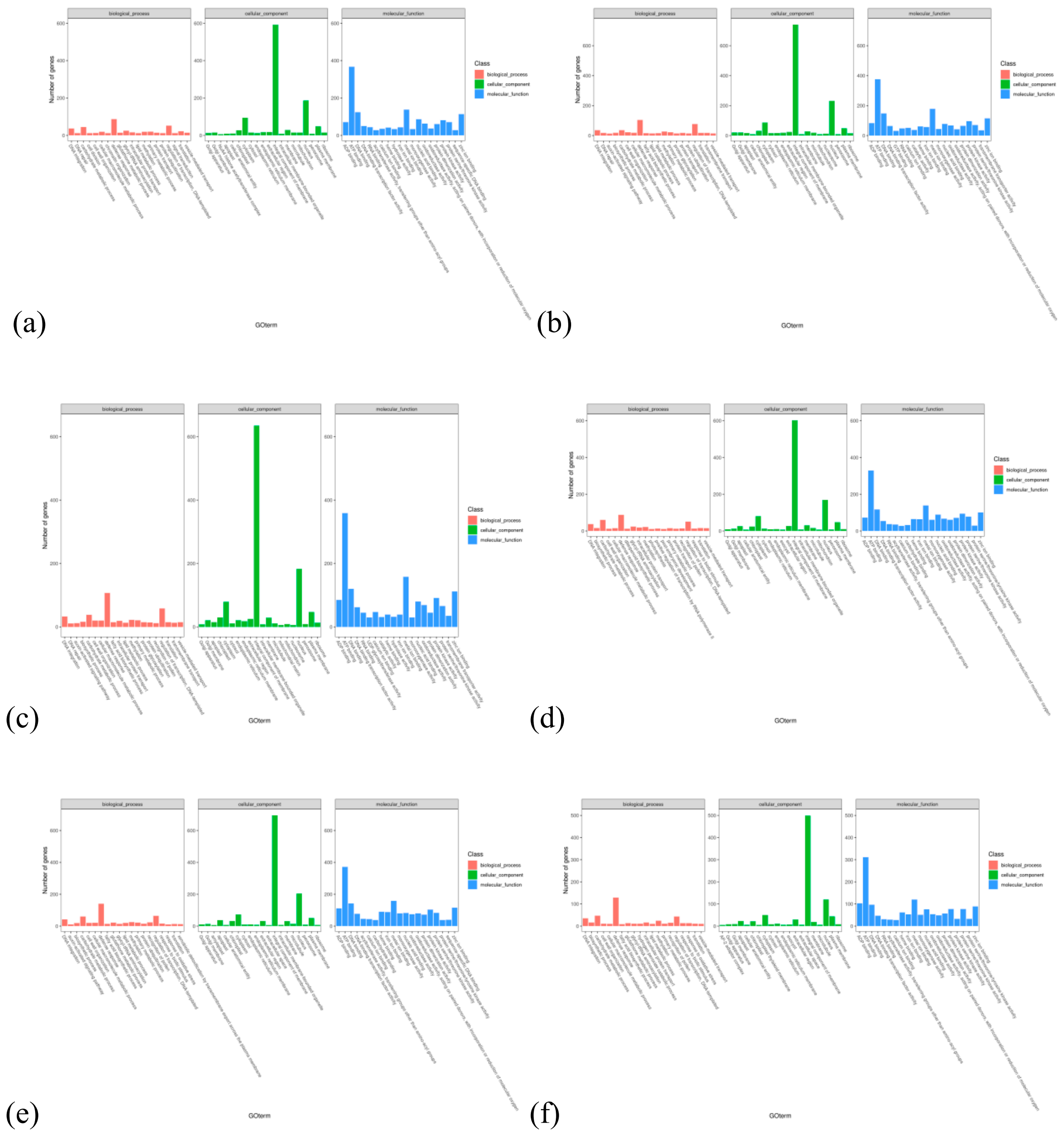

Figure S2. GO enrichment of differentially expressed genes.

(a) GO enrichment analysis in pwf vs ppf; (b) GO enrichment analysis in wwf vs ppf; (c) GO enrichment analysis in wwf vs pwf; (d) GO enrichment analysis in pws vs pps; (e) GO enrichment analysis in wws vs pws; (f) GO enrichment analysis in wws vs pws; GO, gene ontology.
